# The Impact of Hypertension and Related Risk Factors on the Onset and Resolution Rates of Benign Paroxysmal Positional Vertigo Recurrence: A 6-Year Retrospective Study

**DOI:** 10.3390/neurolint17060082

**Published:** 2025-05-25

**Authors:** Alessandro Micarelli, Ivan Granito, Riccardo Xavier Micarelli, Marco Alessandrini

**Affiliations:** 1 Unit of Neuroscience, Rehabilitation and Sensory Organs, UNITER ONLUS, 00153 Rome, Italy; granito.ivan@gmail.com (I.G.); dr.micarelli@gmail.com (R.X.M.); 2 Department of Clinical Sciences and Translational Medicine, ENT Unit, University of Rome Tor Vergata, 00133 Rome, Italy; malessandrini63@gmail.com

**Keywords:** benign paroxysmal positional vertigo, recurrence, hypertension, neuro-otological disorders, aging, hypertension-mediated organ damage, cardiovascular disease

## Abstract

**Background/Objectives:** Due to conflicting results about hypertension and the involvement of associated risk factors in the presentation of idiopathic benign paroxysmal positional vertigo recurrence (R-BPPV), this study aimed to explore possible associations between the resolution rate (RR) and recurrence onset (RO) of R-BPPV, as well as hypertension classification and stages and demographic characteristics. **Methods:** A total of 1201 medical records from patients collected over a 6-year span who first presented with R-BPPV were retrospectively evaluated regarding blood pressure (BP) presentation and associated risk factors. R-BPPV included patients treated with necessary canalith repositioning procedures (CRPs) and followed up with for 12 months. The RO and RR were evaluated when comparing patients sub-grouped by current classification and staging. The association between the RO and RR and many prognostic factors, including the presence of cardio- and neuro-vascular risks, was examined via multiple regression analysis. **Results:** Among the 857 included patients with R-BPPV, 211 presented with an optimal/normal BP, 210 were found to have a high–normal BP, 222 were classified with Grade 1 hypertension, and 214 were found to have Grade 2 hypertension. Significant (*p* < 0.05) progressive earlier presentations and increases in needed CRPs were found with the respective increase in BP subgroups. For the RO, the correlation was statistically significant for age and gender, while for the RR, the correlation was statistically significant for age and hypertension stage. **Conclusions:** This study demonstrates for the first time that clinical consequences of R-BPPV are strongly associated with cardio-, neuro-vascular, and socio-demographic risk factors, which are commonly involved in R-BPPV occurrence.

## 1. Introduction

Benign paroxysmal positional vertigo (BPPV) is a prevalent neuro-otological disorder in clinical settings, exhibiting a 1-year prevalence of 1.6% and a lifetime prevalence of 2.4% [1]. The major pathophysiological process in BPPV involves displaced otoconia from the macula of the utricular otolith, which drop into the semicircular canals [2]. BPPV generally responds well to canalith repositioning procedures (CRPs). However, there is a high recurrence rate after the initial resolution [2], and BPPV recurrence (R-BPPV) is defined as the reappearance of positional vertigo and nystagmus after at least one month following the execution of an effective CRP [3,4]. It was reported that 10% to 18% of patients experience a relapse during their 1-year follow-up period [5], and this relapse rate can be as high as 50% over 10 years [6]. Several studies have demonstrated that delayed BPPV treatment using CRPs, multiple canal involvement, endolymphatic hydrops, bone mineral density, serum vitamin D levels, comorbidities, age, family history, diabetes, and hypertension may be associated with the recurrence of BPPV [2,4,7,8,9,10,11,12]. However, the results are conflicting, especially in the evaluation of the hypertension and associated risk factors [7], including dyslipidemia, impaired glucose tolerance, and type 2 diabetes, which further increase cardiovascular (CV) risk [13,14]. A continuous relationship has been observed between the increase in blood pressure (BP) and the risk of stroke, coronary or ischemic heart disease (CAD), heart failure (HF), as well as the development and progression of chronic kidney disease (CKD). Further, a large number of factors influencing CV risk in patients with hypertension (environmental, lifestyle, and clinical CV risk factors plus hypertension-mediated organ damage [HMOD] and established cardiovascular disease [CVD] or CKD categories) may be involved in R-BPPV presentation [2,3,10,13,14,15]. Thus, current guidelines recommend—in addition to the classification and definition of hypertension grades (which are based on BP values)—to also distinguish stages of hypertension, including HMOD, CVD, CKD, and diabetes [15].

To our knowledge, there are no clinical studies investigating the role of classifying and staging hypertension in BPPV recurrence, so the aim of this study was to explore possible associations between the rate of recurrence of idiopathic BPPV, hypertension classification and stages (including HMOD, CVD, CKD and diabetes), and demographic characteristics, such as age and gender, to determine whether such features could represent a risk factor for the increased probability of recurrence or treatment failure.

## 2. Materials and Methods

### 2.1. Patients, Diagnosis, and Follow-Up

This study is a retrospective evaluation collected over a 6-year span between 2019 and 2024, based on 1201 medical records from patients aged 18 to 99 years (mean age 62.1 ± 14.3 years; 697 females; 504 males) with a first-time diagnosis of R-BPPV, defined as the reappearance of typical symptoms and positional nystagmus after at least 4 weeks from resolution, after previous successful BPPV treatment [3,4]. Patients were referred to the outpatient Otoneurological Service of our Cranio-Cervical and Rehabilitation Institution, after having received in our institution or elsewhere a previous diagnosis of BPPV. The research received ethical approval from the University Hospital Institutional Review Board and complied with the Declaration of Helsinki principles, and all participants gave informed consent following a comprehensive study explanation. The evaluation of R-BPPV patients involved an extensive otoneurological assessment, incorporating pure tone (GSI 61 clinical audiometer, Grason-Stadler, Eden Prairie, MN, USA) and impedance (GSI Tymp Star, Grason-Stadler, USA) audiometry along with video–nystagmoscopic (VisualEyes^TM^, Interacoustics, Middelfart, Denmark) analysis during positioning maneuvers [16,17,18]. According to previous studies, the pure tone threshold was measured in all the participants according to previous procedures [19] and the averaged hearing threshold calculated from four frequencies (500, 1000, 2000, and 4000 Hz; PTA4) following the guidelines of the Committee on Hearing and Equilibrium of the American Academy of Otolaryngology [20]. Thus, patients were categorized as presenting within normal limits (0–20 dB HL) or suffering from mild (21–40 dB HL), moderate (41–70 dB HL), severe (71–89 dB HL), or profound (>90 dB HL) degrees of hearing loss [19]. The diagnosis of R-BPPV involving the posterior semicircular canal was established through the elicitation of direction-changing torsional nystagmus via the Dix–Hallpike maneuver [21]. The nystagmus exhibited a vertical component characterized by an upward rapid phase and a rotational component with a rapid phase directed toward the affected ear, while the BPPV of the lateral semicircular canal manifested as direction-changing horizontal geotropic or apogeotropic nystagmus, which was elicited by the McClure–Pagnini maneuver when the subject was supine and the head was rotated 90 degrees toward the examined ear [22]. According to the direction-changing positional nystagmus features and its hypothesized pathophysiology [23,24], LSC BPPV has been categorized as canalolithiasis (ca-LSC) or cupulolithiasis (cu-LSC). The anterior semicircular canal (ASC) BPPV has been characterized by positional nystagmus exhibiting downward movements, accompanied by a slight torsional geotropic or non-geotropic component during Dix–Hallpike CRP [16]. All patients underwent CRPs (Epley, Baloh, and ‘‘reverse’’ Epley maneuvers for PSC, LSC, and ASC BPPV, respectively, [25]) and were re-tested 7 days later. Individuals exhibiting persistent symptoms and nystagmus underwent further CRPs and were subsequently recalled for additional examinations [16]. Patients were followed up for 12 months. In order to avoid the likelihood of residual dizziness, positional vertigo with nystagmus was considered when diagnosing a true R-BPPV and/or approaching subsequent CRPs [26]. All patients were evaluated with an accurate clinical history, including standard questions about associated diseases and pharmacological therapies, as well as history of previous BPPV episode clinical characteristics, and routine blood samples. Subjects with significant risk factors related to non-idiopathic BPPV and/or R-BPPV, including but not limited to endocrine disorders (excluding diabetes), osteoporosis, psychiatric conditions, autoimmune diseases, vitamin D deficiency, and trauma, were omitted from the study [27,28,29,30,31,32]. Participants with prior neuro-otological conditions, such as Menière’s disease, or those on medications potentially influencing BPPV and/or the onset of R-BPPV were excluded from the study [33]. Patients with incomplete clinical history, or “persistent” BPPV, namely no remission of symptoms or nystagmus after 2 weeks or five repositioning maneuvers [34], were excluded. Cerebral magnetic resonance imaging (MRI) was performed in order to exclude neurological diseases and/or malformations [16]. The classification and stages of hypertension were defined according to the 2023 ESH guidelines [15] as follows: Classification: optimal (systolic blood pressure [SBP] < 120 mmHg and diastolic blood pressure [DBP] < 80 mmHg), normal (SBP = 120–129 mmHg and DBP = 80–84 mmHg), high–normal (SBP = 130–139 mmHg and DBP = 85–89 mmHg), Grade 1 (SBP = 140–159 mmHg and DBP = 90–99 mmHg), or Grade 2 (SBP = 160–179 mmHg and DBP = 100–109 mmHg). Staging: Stage 1: Uncomplicated hypertension (i.e., without HMOD or established CVD, but including CKD stage 1 and 2); Stage 2: Presence of HMOD or CKD stage 3 or diabetes; Stage 3: Established CVD or CKD stages 4 or 5. Grade 3, isolated systolic and diastolic hypertension were not included in this study due to the paucity of cases, and optimal and normal BP classes were merged together. BP measurement was established upon office blood pressure monitoring (OBPM) or home blood pressure monitoring (HBPM) in incidental and stable hypertension, respectively, according to the current guidelines [15,35,36]. Patients’ blood pressure treatment was evaluated as lifestyle changes, mono-, or poly-treatment. Patients with a first hypertension diagnosis received appropriate medications and were followed up with. For patients aware of hypertension due to recent incidental OBPM who were awaiting a proper treatment, or due to HBPM who were awaiting further clinical assessment and treatment, a definite diagnosis with treatment and follow-up was achieved. In order to avoid bias related to demographic and BP-related presentation, patient enrollment was performed with a stratified random sampling, including BP classification, age, BMI, and gender [37].

Following previous experiences—beyond registry details (age and gender) and hypertension classification and staging—clinical characteristics of R-BPPV were thus considered, including the recurrence onset (RO), number of maneuvers needed to achieve resolution (resolution rate, RR), and involved canal [33,38].

### 2.2. Data Handling and Statistical Analysis

To estimate the characteristics of the study sample, our statistical approach consisted of means, frequencies, and percentages. The X^2^ test was carried out to define associations between categorical factors, including treatment options for high blood pressure and hearing loss categories and groups. To assess whether the data were of Gaussian distribution, D’Agostino K-squared normality and Levene’s homoscedasticity test were applied (where the null hypothesis is that the data are normally and homogenously distributed) [39]. According to previous procedures [38], the RO was categorized as follows: 2–4 weeks = 1; 4–6 weeks = 2; 6–8 weeks = 3; >8 weeks = 4. The RR was categorized as follows: 1 maneuver = 1; 2 maneuvers = 2; 3 maneuvers = 3; >4 maneuvers = 4. Gender was scored as 0 for male and 1 for female. The categorization of canal involvement was 1 for PSC, 2 for ca-LSC, 3 for cu-LSC, and 4 for multicanal presentation. Finally, subjects’ BP classification was subgrouped into several classes: 1 for high normal, 2 for Grade 1 hypertension, and 3 for Grade 2 hypertension; subjects’ hypertension stages were classified as follows: 1 for stage 1, 2 for stage 2, and 3 for stage 3. The values 0 and 1 were used to, respectively, categorize the absence or presence of smoking habits (with a yes/no question regarding current smoking status), type II diabetes, CKD, non-high-density lipoprotein, and HMOD and CVD [15]. Given the exploratory nature of the study, as well as previous biomedical retrospective approaches [40], the association between the RO and RR and all the prognostic factors (the canal involved [including PSC, ca-LSC, cu-LSC, and multicanal presentation], age, BMI, gender, and hypertension classification and stages, as well as the presence of smoking habits, type II diabetes, CKD, non-high-density lipoprotein, and HMOD and CVD) was examined via multiple regression analysis in patients with R-BPPV affected with high–normal, Grade 1, or Grade 2 hypertension (*n* = 646). *p*-values less than 0.05 were considered statistically significant [41].

## 3. Results

**Figure 1:**
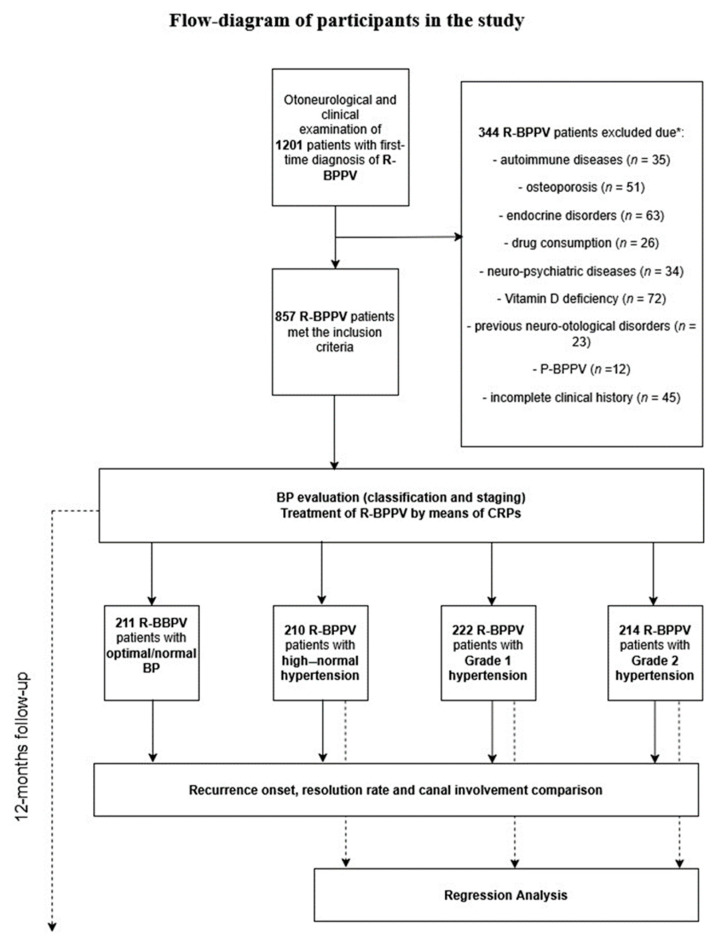
Flow diagram of participants in the study. P-BPPV, persistent benign paroxysmal positional vertigo; R-BPPV, recurrent benign paroxysmal positional vertigo; CRPs, canalith repositioning procedures; BP, blood pressure; *, participants excluded due to the presence of more than one comorbidity.

Among 1201 patients, 344 were excluded due to the presence of risk factors significantly associated with nonidiopathic forms of BPPV or R-BPPV (details in Figure 1).

The remaining 857 (mean age = 61.3 ± 15.9 years; 514 females, 343 males) patients with R-BPPV were treated with specific repositioning maneuvers and reassessed every 7 days until their symptoms resolved. In this group, 211 patients (mean age = 60.7 ± 15.9 years; 126 females, 85 males) presented with an optimal/normal BP, 210 patients (mean age = 60.6 ± 15.9 years; 129 females, 81 males) were found to have a high–normal BP, 222 patients (mean age = 60.9 ± 15.7 years; 134 females, 88 males) were classified with Grade 1 hypertension, and 214 patients (mean age = 62.9 ± 16.05 years; 125 female, 89 males) were found to have a Grade 2 hypertension (Table 1, Figure 1).

Among 502 patients presenting with R-BPPV of PSC, 212 (42.2%), 138 (27.4%), 90 (17.9%), and 62 (12.35%) had a recurrence onset of, respectively, 4–6 weeks, 6–8 weeks, 8–10 weeks, and >10 weeks. A total of 132 (26.2%), 187 (37.2%), 121 (24.1%), and 62 (12.4%) needed, respectively, 1, 2, 3, or more than 4 CRPs. Of those with ca-LSC R-BPPV (*n* = 273), 96 (35.1%), 88 (32.2%), 56 (20.5%), and 33 (12%) had a recurrence onset of 4–6 weeks, 6–8 weeks, 8–10 weeks, and >10 weeks, respectively. A total of 62 (22.7%), 107 (39.1%), 74 (27.1%), and 30 (10.9%) needed, respectively, 1, 2, 3, or more than 4 CRPs. Among 49 participants complaining of cu-LSC R-BPPV, 19 (38.7%), 11 (22.4%), 16 (32.6%), and 3 (6.1%) reported a recurrence onset of, respectively, 4–6 weeks, 6–8 weeks, 8–10 weeks, and >10 weeks. Among the same patients, 4 (8.1%), 20 (40.8%), 17 (34.6%), and 8 (16.3%) needed 1, 2, 3, or more than 4 CRPs, respectively. Finally, of the participants with multicanal presentation (*n* = 33), 18 (54.5%), 7 (21.2%), 6 (18.1%), and 2 (6%) had a recurrence onset of 4–6 weeks, 6–8 weeks, 8–10 weeks, and >10 weeks, respectively, and 9 (27.2%), 9 (27.2%), 8 (24.2%), and 7 (21.2%) needed 1, 2, 3, or more than 4 CRPs, respectively. Associations between canal presentation were found to be not significant for neither the recurrence onset (X^2^ = 14.2; *p* = 0.11) nor the resolution rate (X^2^ = 13.1; *p* = 0.15).

**Figure 2:**
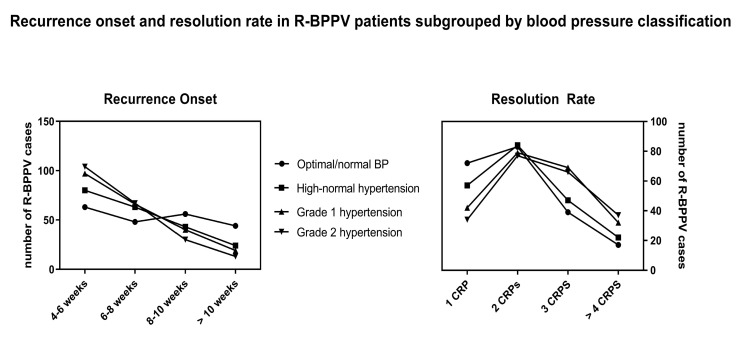
Differences in recurrence onset and resolution rate in patients with recurrent benign paroxysmal positional vertigo (R-BPPV) when subgrouped by blood pressure (BP) classification. CRPs, canalith repositioning procedures.

Although there were no significant differences in canal presentation among the four groups (Table 1), a significant progressive earlier presentation was found during the 12-month follow-up (i.e., decrease in RO; X^2^ = 45.35, *p* < 0.05), along with an increase in the needed repositioning maneuvers (i.e., increase in RR; X^2^ = 37.42, *p* < 0.05) corresponding to the respective increase in BP subgroups (Table 1, Figure 2). Among 646 patients with high–normal to Grade 2 hypertension, regarding the RO, 44 (15.7%), 67 (23.9%), and 169 (60.3%) were experiencing recurrence after 4–6 weeks (*n* = 280) while receiving lifestyle changes, monotherapy, or polytherapy, respectively. Similarly, 38 (19.3%), 59 (30.1%), and 99 (50.5%) patients experienced recurrence after 6–8 weeks (*n* = 196); 24 (21.2%), 34 (30%) and 55 (48.6%) patients receiving lifestyle changes, monotherapy, or polytherapy experienced recurrence after 8–10 weeks (*n* = 113), respectively; and 13 (22.8%), 19 (33.3%), and 25 (43.8%) patients, respectively, belonging to the same category were found to present with R-BPPV after 10 weeks (*n* = 57). With regard to RR, 32 (24.2%), 35 (26.5%), and 65 (49.2%) patients, respectively, who were receiving lifestyle changes, monotherapy, or polytherapy needed 1 CRP (*n* = 132). Among those needing 2 CRPs (*n* = 241), 51 (21.1%), 61 (25.3%), and 129 (53.5%) were receiving lifestyle changes, monotherapy, or polytherapy, respectively. A total of 27 (14.8%), 52 (28.5%), and 103 (56.5%) patients belonging, respectively, to the same category, were found to need 3 CRPs (*n* = 182). Then, 9 (9.8%), 31 (34%), and 51 (56%) patients, respectively, receiving lifestyle changes, monotherapy, or polytherapy needed more than 4 CRPs (*n* = 91). No significant association was found between treatment options and the RO (X^2^ = 9.25; *p* = 0.159932) and RR (X^2^ = 11.08; *p* = 0.08). Among 857 patients ranging from optimal/normal to Grade 2 hypertension, 375 (43.7%) presented within normal limits (10.8 ± 5.6 dB HL), 230 (26.8%) suffered from a mild degree of hearing loss (30 ± 6.2 dB HL), 173 (20.1%) suffered from a moderate degree of hearing loss (57.3 ± 8.5 dB HL), 55 (6.4%) suffered from a severe degree of hearing loss (78.9 ± 5.6 dB HL), and 24 (2.8%) suffered from a profound degree of hearing loss (99.9 ± 6.7 dB HL). Table 2 depicts the relationship between the RO and RR across various degrees of hearing loss, showing a slight—yet significant—increase in the number of patients experiencing mild and moderate hearing loss who present with R-BPPV after 4 and 8 weeks, respectively, and who require at least 2 CRPs to achieve resolution.

Multiple regression analysis was conducted for all patients with R-BPPV classified by high–normal to Grade 1 and Grade 2 (*n* = 646, mean age = 61.4 ± 15.9 years; 388 females, 258 males) (clinical data are presented in Table 3) to determine the RO and RR in relation to the main prognostic factors (gender, age, canal, grading, stage, smoking habits, diabetes, CKD, HMOD, CVD, and non-HDL and BMI).

**Figure 3:**
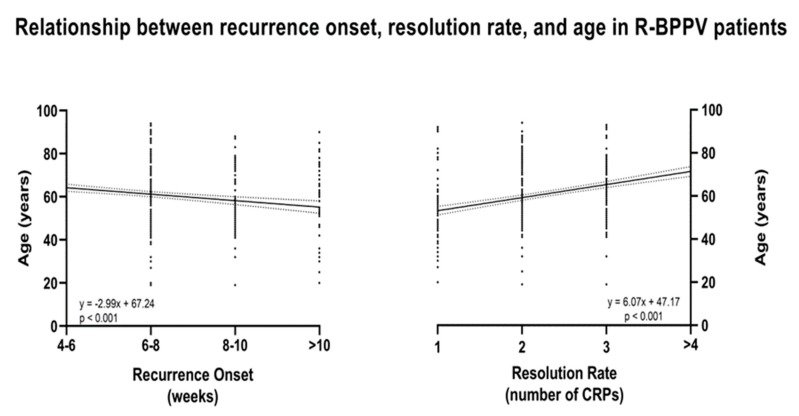
Regression plot depicting the relationship between resolution rate, recurrence onset, and age in recurrent benign paroxysmal positional vertigo (R-BPPV).

For RO, the correlation was only statistically significant for age and gender with partial correlation coefficients, respectively, of −0.15 and 0.09 (Table 4, Figure 3), while for RR, the correlation was statistically significant for age and stage with partial correlation coefficients of −0.33 and 0.19 (Table 4, Figure 3), respectively.

## 4. Discussion

The first interesting finding of the present study is the progressive significant increase in RR and decrease in RO across the different classes of hypertension, from optimal/normal to Grade 2 hypertension (Table 3, Figure 2), with no significant associations with different treatment options.

These findings are relevant considering that, on one hand—despite age and gender—this retrospective study tried to isolate hypertension and related/descending risk factors from those further established to be involved in the recurrence of R-BPPV [1,2,4,7,8,9,10,11,12,13]. On the other hand, several previous studies have suggested that ischemic vascular changes—possibly involved in hypertension—may contribute to the pathophysiology of otoconial dislodgement [42,43] due to tissue hypoxia and cochleovestibular degeneration [44,45,46]. The vestibular system’s vascular supply, derived from the anterior inferior cerebellar artery, which branches into the anterior vestibular artery, is susceptible to ischemic obstruction due to its limited collateral supply [46]. Therefore, ischemic alterations in the vertebrobasilar system may initially manifest as vestibular disorders, such as BPPV [46]. A few retrospective studies have indicated a greater prevalence of cardiovascular diseases, particularly hypertension, among patients with BPPV compared to control groups [46,47]. However, carotid artery intima-media thickness is increased in individuals with BPPV compared to those with other peripheral vestibular disorders [48] and in those with hypertension [49]. This may reinforce the data of the present study and postulate for the first time that the occurrence of R-BPPV and its severity in terms of the RR and RO may be fostered—in addition to other risk factors—by aforementioned atherosclerotic changes induced by hypertension [49,50,51]. This could be highlighted by the slight increase in frequency presentation of R-BPPV between the 8th and 10th week and after 10 weeks in patients with optimal/normal BP and relative anticipation of recurrence before such timepoints in patients with R-BPPV affected by hypertension. This aspect could be related to the above-mentioned general cochleovestibular degeneration related to tissue hypoxia due to atherosclerotic changes involving patients affected by different stages of hypertension. It could encourage clinical practitioners to strictly follow up with these patients, especially during the first two months. Further, patients in this retrospective study were also staged, including all comorbidities associated with and/or descending from increased BP [15]. This was due to the relationship between BP increase and HMOD, diabetes, CVD, and CKD [15], finally impacting on atheroscletoric changes [49,51] and may be used to more accurately stage patients suffering from hypertension. When studying all the patients with BP increase as a continuum by means of the regression analysis, also including hypertension stages, it has been found for the first time that the RR increases with stage increasing, finally impacting R-BPPV severity (Table 4). This aspect is noteworthy since it may lead to the hypothesis that the hypertension stage involves vascular damage, ischemia, or atherosclerosis induced by vascular comorbidities (including hypertension). Further, it could be hypothesized that it could cause displacement or degenerative changes in otoconia [1,46,52], not only resulting in the recurrence of BPPV but also in its clinical consequences in terms of resolution. It has been further found that RO and RR were—respectively—positively and negatively associated with age (Table 4, Figure 3). This aspect is still debated in the literature [1]; such findings not only confirm previous studies highlighting that the risk of recurrence increased with age [2,53,54,55] but evidence for the first time that R-BPPV severity may be further impacted by age in patients with hypertension. This phenomenon may be explained by the fact that otoconial detachment tends to increase with age [54] and becomes more relevant since the patients’ large-scale samples were smoothed—during the enrollment process—from confounding risk factors for R-BPPV occurrence, which are known to increase with age [1,2,13]. Finally, the present study found that female gender is associated with a decrease in RO (Table 4), thus reducing the time free from disease, possibly due to hormonal changes finally impacting on otoconial demineralization [1,2,56,57].

Collaterally, although some studies evidenced a moderate correlation between the worse hearing ear in patients with BPPV [58], no studies exist that have further deepened this aspect in R-BPPV beyond the association with Meniere’s disease [59]. The findings of the present study (Table 2) found a slight but significant increase in the number of patients experiencing mild to moderate hearing loss and presenting with R-BPPV after, respectively, 4 and 8 weeks, who required at least 2 CRPs to achieve resolution. However, these data may be biased due to the spontaneous degeneration of the vestibulocochlear system in the overrepresented elderly population of this study, where a higher prevalence of hearing loss is present [60].

In conclusion, since the recurrence of BPPV may result in poor quality of life, vertigo, and dizziness, thereby increasing the risk of falls, it is relevant not only to understand the risk factors for the recurrence of BPPV but also to deepen our understanding of these factors, in hopes of possibly reducing the resolution after CRPs [56]. This study demonstrated for the first time that clinical consequences of R-BPPV have a strong association with cardio-, neuro-vascular, and socio-demographic risk factors commonly involved in the occurrence of R-BPPV. This study suggests that clinical manifestations of R-BPPV may be influenced by cardio-, neuro-vascular, and socio-demographic risk factors, which are also commonly associated with its recurrence [46,47,48]. These risk factors are a worldwide burden [61], and—aside from needing further studies—the findings of the present large-scale study encourage a deeper exploration of these aspects to confirm these associations and clarify their clinical implications, ultimately improving the clinical treatment of R-BPPV.

### Limitations of the Study

The findings of the present study must be interpreted with caution due to the presence of some limitations. First of all, the absence of vascular imaging, such as Doppler ultrasound of the extracranial arteries, which could have strengthened direct evidence of atherosclerotic burden involved in R-BPPV, has already been used in the literature [62]. Future studies should consider including vascular imaging to better characterize the role of microvascular dysfunction in R-BPPV pathogenesis. Secondly, the hormonal status of female participants, including menopausal status, was not systematically assessed. Therefore, the potential impact of hormonal changes and menopause [63] on the outcomes observed could not be fully evaluated. This limitation is acknowledged, and future studies will include a more detailed assessment of hormonal status and its possible influence on the results. Finally, in line with previous literature [38], the number of affected canals was considered a discrete numerical variable (range: 1–4), reflecting an actual count rather than a categorical classification. This approach aligns with methodological recommendations in the literature, which suggest treating ordinal or discrete numerical variables as such in statistical analyses, unless there is a specific rationale for categorical coding [64].

## Figures and Tables

**Table 1:** Clinical and demographic presentation of recurrent benign paroxysmal positional vertigo patients subgrouped according to blood pressure classification.

BP Classification	Age (Mean ± SD)		Gender		X^2^
			M	F	
**Optimal/normal (*n* = 211)**	60.79 ± 15.95		85 (40.28%)	126 (59.71%)	X^2^ = 0.42; *p* = 0.93
**High–normal (*n* = 210)**	60.64 ± 15.91		81 (38.57%)	129 (61.42%)	
**Grade 1 (*n* = 222)**	60.91 ± 15.74		88 (39.46%)	134 (60.08%)	
**Grade 2 (*n* = 214)**	62.9 ± 16.05		89 (41.58%)	125 (58.41%)	
**Recurrence Onset (weeks)**					
	***4–6***	***6–8***	***8–10***	***>10***	
**Optimal/normal (*n* = 211)**	63 (29.85%)	48 (22.74%)	56 (26.54%)	44 (20.85%)	X^2^ = 45.35; *p* < 0.05
**High–normal (*n* = 210)**	80 (38.09%)	63 (30%)	43 (20.47%)	24 (11.42%)	
**Grade 1 (*n* = 222)**	97 (43.49%)	66 (29.59%)	40 (17.93%)	19 (8.52%)	
**Grade 2 (*n* = 214)**	104 (48.59%)	67 (31.3%)	30 (14.01%)	13 (6.07%)	
**Resolution Rate (number of CRPs)**					
	***1***	***2***	***3***	***>4***	
**Optimal/normal (*n* = 211)**	72 (34.12%)	83 (39.33%)	39 (18.48%)	17 (8.05%)	X^2^ = 37.42; *p* < 0.05
**High–normal (*n* = 210)**	57 (27.14%)	84 (40%)	47 (22.38%)	22 (10.47%)	
**Grade 1 (*n* = 222)**	42 (18.83%)	79 (35.42%)	69 (30.94%)	32 (14.34%)	
**Grade 2 (*n* = 214)**	34 (15.88%)	77 (35.98%)	66 (30.84%)	37 (17.28%)	
**Canal**					
	***PSC***	***ca-LSC***	***cu-LSC***	***Multicanal***	
**Optimal/normal (*n* = 211)**	125 (59.24%)	68 (32.22%)	12 (5.68%)	6 (2.84%)	X^2^ = 2.74; *p* = 0.97
**High–normal (*n* = 210)**	127 (60.47%)	66 (31.42%)	9 (4.28%)	8 (3.8%)	
**Grade 1 (*n* = 222)**	130 (58.29%)	69 (30.94%)	13 (5.82%)	10 (4.48%)	
**Grade 2 (*n* = 214)**	120 (56.07%)	70 (32.71%)	15 (7%)	9 (4.2%)	

Table 1: Clinical and demographic presentation of 857 patients with recurrent benign paroxysmal positional vertigo according to blood pressure classification. The following abbreviations are used: CRPs, canalith repositioning procedures; PSC, posterior semicircular canal; LSC, lateral semicircular canal; ca, canalolithiasis; and cu, cupulolithiasis.

**Table 2:** Association between hearing loss, recurrence onset, and resolution rate in 857 patients with R-BPPV.

	Normal Limit (0–20 dB)	Mild HL (21–40 dB)	Moderate HL (41–70 dB)	Severe HL (71–89 dB)	Profound HL (>90 dB)	X^2^
Recurrence Onset						
4–6 weeks (*n* = 343)	167 (48.6%)	67 (19.5%)	80 (23.3%)	22 (6.4%)	7 (2.04%)	42.8 *p* < 0.001
6–8 weeks (*n* = 244)	102 (41.8%)	65 (26.6%)	61 (25%)	13 (5.3%)	3 (1.2%)	
8–10 weeks (*n* = 169)	65 (38.4%)	61 (36%)	20 (11.8%)	14 (8.2%)	9 (5.3%)	
>10 weeks (*n* = 101)	41 (40.5%)	37 (36.6%)	12 (11.8%)	6 (5.9%)	5 (4.95)	
Resolution Rate						
1 CRP (*n* = 204)	79 (38.7%)	54 (26.4%)	44 (21.5%)	19 (9.3%)	8 (3.9%)	31.05 *p* = 0.001
2 CRPs (*n* = 324)	134 (41.3%)	112 (34.5%)	51 (15.7%)	17 (5.2%)	10 (3%)	
3 CRPs (*n* = 221)	107 (48.4%)	48 (21.7%)	49 (22.1%)	13 (5.8%)	4 (1.8%)	
>4 CRPs (*n* = 108)	55 (50.9%)	16 (14.8%)	29 (26.8%)	6 (5.5%)	2 (1.85%)	

Table 2: The association between degrees of hearing loss (HL), recurrence onset, and resolution rate in 857 patients with recurrent benign paroxysmal positional vertigo (R-BPPV).

**Table 3:** Risk factors presentation in 646 patients with recurrent benign paroxysmal positional vertigo affected by hypertension.

BP Classification	Systolic BP (Mean ± SD)	Diastolic BP (Mean ± SD)	Staging	Smoking Habits	Diabetes	CKD Stage	HMOD	CVD	Non-HDL	BMI (Mean ± SD)
**High–normal (*n* = 210)**	134.56 ± 2.74	87.04 ± 1.41	2; *n* = 29 1; *n* = 181	*n* = 95	*n* = 12	3; *n* = 7 2; *n* = 17 1; *n* = 186	*n* = 21	*n* = 0	*n* = 17	26.87 ± 1.71
**Grade 1 (*n* = 222)**	149.68 ± 5.59	94.57 ± 2.92	3; *n* = 11 2; *n* = 42 1; *n* = 169	*n* = 105	*n* = 26	4; *n* = 6 3; *n* = 19 2; *n* = 17 1; *n* = 180	*n* = 36	*n* = 10	*n* = 27	27.53 ± 1.78
**Grade 2 (*n* = 214)**	169.48 ± 5.69	104.2 6 ± 2.88	3; *n* = 25 2; *n* = 48 1; *n* = 141	*n* = 103	*n* = 41	5; *n* = 4 4; *n* = 17 3; *n* = 24 2; *n* = 22 1; *n* = 147	*n* = 56	*n* = 22	*n* = 32	27.98 ± 1.98

Table 3: Presentation of risk factors in 646 patients affected by recurrent benign paroxysmal positional vertigo presenting with hypertension. BP, blood pressure; SD, standard deviation; chronic kidney disease, CKD; hypertension-mediated organ damage, HMOD; cardiovascular disease, CVD; high-density lipoprotein, HDL; body mass index, BMI.

**Table 4:** Multiple regression model of the resolution rate in relation to prognostic factors in patients with R-BPPV.

	Partial Regression Coefficient	Std. Err	t	*p*-Value	Cnf. Lmt −95.00%	Cnf. Lmt +95.00%	Partial Correlation Coefficient (ß)	Std. Err. ß	Cnf. Lmt −95.00%	Cnf. Lmt +95.00%
	**Recurrence Onset**									
Intercept	3.4	0.65	5.19	**<0.001**	2.11	4.68				
Age	−0.009	0.002	−4.08	**<0.001**	−0.01	-0.005	−0.15	0.03	−0.23	−0.08
Gender	0.18	0.08	2.13	**0.03**	0.01	0.35	0.09	0.04	0.007	0.17
BPPV Canal	0.01	0.04	0.39	0.69	−0.07	0.11	0.01	0.03	−0.05	0.09
Grading	−0.07	0.04	−1.42	0.15	−0.16	0.02	−0.05	0.04	−0.13	0.02
Stage	−0.21	0.17	−1.22	0.21	−0.55	0.12	−0.12	0.1	−0.32	0.07
Smoking Habits	−0.004	0.07	−0.06	0.95	−0.15	0.14	−0.002	0.03	−0.07	0.07
Diabetes	−0.05	0.15	−0.36	0.71	−0.36	0.25	−0.01	0.05	−0.12	0.08
CKD Stage	−0.04	0.07	−0.59	0.55	−0.19	0.1	−0.03	0.06	−0.16	0.08
HMOD	−0.11	0.13	−0.87	0.38	−0.38	0.14	−0.04	0.05	−0.14	0.05
CVD	0.35	0.24	1.42	0.15	−0.13	0.84	0.07	0.05	−0.02	0.18
Non-HDL	−0.13	0.12	−1.07	0.28	−0.37	0.11	−0.04	0.04	−0.12	0.03
BMI	−0.01	0.02	−0.83	0.4	−0.06	0.02	−0.03	0.04	−0.12	0.05
	**Resolution Rate**									
Intercept	0.47	0.58	0.8	**<0.001**	−0.68	1.62				
Age	0.02	0.002	9.44	**<0.001**	0.01	0.02	0.33	0.03	0.26	0.4
Gender	0.01	0.07	0.25	0.79	−0.13	0.17	0.01	0.03	−0.06	0.08
BPPV Canal	0.05	0.04	1.34	0.18	−0.02	0.14	0.04	0.03	−0.02	0.11
Grading	0.04	0.04	0.94	0.34	−0.04	0.12	0.03	0.03	−0.03	0.1
Stage	0.32	0.15	2.08	**0.03**	0.01	0.63	0.19	0.09	0.01	0.37
Smoking Habits	−0.1	0.06	−1.57	0.11	−0.24	0.02	−0.05	0.03	−0.12	0.01
Diabetes	0.08	0.14	0.59	0.55	−0.19	0.36	0.02	0.04	−0.06	0.12
CKD Stage	0.06	0.06	1	0.31	−0.06	0.2	0.05	0.05	−0.05	0.17
HMOD	0.08	0.12	0.68	0.49	−0.15	0.32	0.03	0.04	−0.06	0.12
CVD	0.22	0.22	1.02	0.3	−0.2	0.66	0.05	0.05	−0.04	0.15
Non-HDL	−0.03	0.11	−0.29	0.76	−0.25	0.18	−0.01	0.03	−0.08	0.06
BMI	−0.0002	0.02	−0.01	0.99	−0.04	0.04	−0.0004	0.04	−0.08	0.08

Table 4: Multiple regression model of the recurrence onset and resolution rate in relation to prognostic factors in 646 patients with recurrent benign paroxysmal positional vertigo (R-BPPV) affected by hypertension. Benign paroxysmal positional vertigo, BPPV; Standard, Std; error, Err; confidence, Cnf; limit, Lmt; SD, standard deviation; chronic kidney disease, CKD; hypertension-mediated organ damage, HMOD; cardiovascular disease, CVD; high-density lipoprotein, HDL; body mass index, BMI. In bold, significant *p*-values.

## Data Availability

Data are unavailable due to privacy and ethical restrictions.
